# Functionalization of Oligosiloxane for Polyester Comonomer

**DOI:** 10.3390/molecules30132775

**Published:** 2025-06-27

**Authors:** Satoru Saotome, Jiaorong Kuang, Reina Akashi, Momoko Takahashi, Yujia Liu, Takayuki Iijima, Masafumi Unno

**Affiliations:** 1 Department of Chemistry and Chemical Biology, Gunma University, 1-5-1 Tenjin-cho, Kiryu 376-8515, Gunma, Japan; satoru.saotome.ma@mcgc.com (S.S.); t242a002@gunma-u.ac.jp (J.K.); t221a003@gunma-u.ac.jp (R.A.); t221a064@gunma-u.ac.jp (M.T.); 2 Polyester Laboratory, Polymers Research & Development Center, Mitsubishi Chemical Corporation, 1, Toho-cho, Yokkaichi-shi 510-8530, Mie, Japan; takayuki.iijima.ma@mcgc.com

**Keywords:** siloxane, polyester, copolymerization, hydrophobic surface, low-temperature impact resistance

## Abstract

This paper proposes a new functionalized oligosiloxane as a comonomer for polyester, designed to provide hydrophobic surface properties and enhance low-temperature impact resistance. The functionalization of polymer resin itself has attracted attention in the context of monomaterialization. Chemically designing the primary structure of not only polymers but also monomers is crucial for enhancing the intrinsic performance of the resin. However, little is known about oligosiloxane monomers for polyester that can provide oligosiloxane-like properties such as hydrophobicity and flexibility at low temperatures. Here, we report the functional design of a polyester material through silicone copolymerization. A novel comonomer was designed and synthesized to optimize both the molecular structure and the compatibility of the silicone segments, promoting uniform copolymer formation. Incorporating silicone into the polymer matrix reduced surface energy, thereby improving water repellency. Furthermore, the flexibility imparted by the silicone components effectively mitigated the brittleness of polyester at sub-zero temperatures, resulting in superior impact resistance. Structural analysis, contact angle measurements, and low-temperature impact tests were conducted on the copolymers. The results confirmed that optimizing comonomer design enables significant enhancement of both hydrophobicity and impact durability, contributing to the development of high-performance polyester materials suitable for demanding environments.

## 1. Introduction

Polyesters are widely used in various industries, including textiles, packaging, and engineering applications, due to their excellent mechanical properties, processability, and thermal stability. However, their surfaces often exhibit hydrophilic characteristics, leading to limitations in water resistance and contamination prevention [1,2]. Recently, as the push for monomaterialization has intensified, there has been a growing demand for the development of coating-free materials by directly imparting functionality to plastics. From this perspective, achieving hydrophobization of polyester surfaces is a crucial aspect of material design.

Siloxane copolymerization has emerged as a promising strategy for modifying polymer surfaces, as siloxanes possess inherently low surface energy and excellent water-repellent properties [3,4,5]. The incorporation of siloxane into polyester structures offers the potential to significantly improve hydrophobicity while maintaining the desirable mechanical properties of the base polymer. Furthermore, siloxane provides flexibility to the polymer, which could enhance impact resistance, particularly in low-temperature environments. However, although several studies have explored siloxane copolymerization with polyesters, a key challenge remains: achieving uniform copolymerization while fully harnessing the advantageous characteristics of siloxanes [6,7]. Previous approaches have often resulted in phase separation or poor compatibility, hindering optimal material performance and limiting practical applications.

The difficulty in achieving homogeneous copolymerization arises from the intrinsic differences in polarity between polyesters and siloxanes. Polyesters generally possess high polarity, whereas siloxanes exhibit low polarity, making uniform incorporation challenging [8,9,10]. This incompatibility often leads to bleed-out of siloxane to the surface, decreasing the intended hydrophobic effects [11,12]. Addressing this issue requires advancements in molecular architecture to improve the compatibility between these two components.

In this study, we focus on the molecular design and synthesis of novel siloxane comonomers specifically tailored for polyester copolymerization. By optimizing the chemical structure and compatibility of these comonomers, we aim to facilitate seamless integration into the polyester matrix, promoting uniform copolymer formation and enhancing surface hydrophobicity. The synthesized copolymers are evaluated through structural characterization, contact angle measurements, and mechanical testing under various conditions. We investigate the influence of siloxane incorporation on both the surface properties and low-temperature impact resistance of the polyester material.

## 2. Results and Discussion

### 2.1. Synthesis of Siloxane-Based Comonomers

**Scheme 1:**
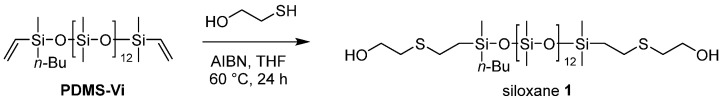
Synthesis of Siloxane **1**.

**Scheme 2:**
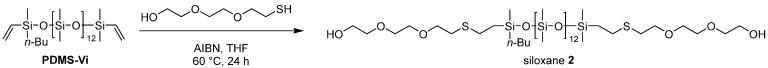
Synthesis of Siloxane **2**.

**Scheme 3:**
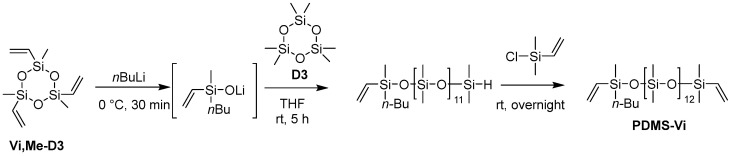
Synthesis of PDMS-Vi.

The targeted polyester-friendly siloxane-based comonomers were designed to slightly increase the polarity of the siloxane. Specifically, oligosiloxanes modified with oligoethyleneoxides at both termini (Scheme 1 and Scheme 2) were synthesized. The starting compound PDMS-Vi was synthesized by ring opening polymerization (ROP) of hexamethylcyclotrisiloxane (D3) using an appropriate initiator and end-capping agent (Scheme 3). The degree of polymerization was adjusted so that each molecule contained 14 silicon atoms. Short siloxane chains cannot achieve hydrophobicity and low-temperature impact resistance because polysiloxane forms one helix per six silicone atoms [13,14,15]. On the other hand, long siloxane chains deteriorate compatibility with polyester, as the long siloxane chains reduce the overall molecular polarity [16,17,18].

The thiol-ene reaction was adopted as a strategy for introducing oligoethyleneoxides into oligosiloxanes [19,20]. Herein, we synthesized ethanol-modified siloxane 1 and dioxyethylene ethanol-modified siloxane 2.

**Figure 1:**
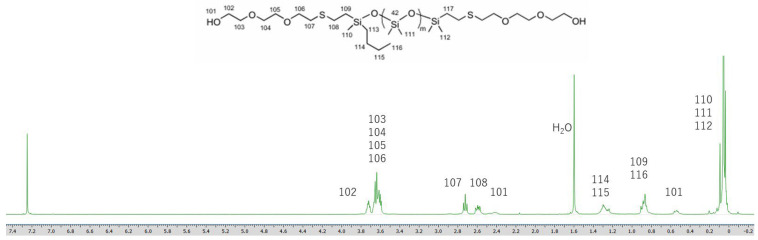
^1^H NMR spectrum of siloxane **2**.

The structures of the obtained oligosiloxanes were confirmed through MALDI-TOF MS measurements (Figures S11–S13). The target siloxane, PDMS-Vi (n = 12), was synthesized via the ring-opening polymerization of the D3 monomer. In addition to the main product (n = 12), byproducts with different degrees of polymerization, including siloxanes with n = 9, 15, 18, and 21, were also observed (Figure S11). The corresponding thiolated byproducts are also present in the MALDI-TOF-MS spectra of siloxane 1 and 2 (Figures S12 and S13). Based on the integration of the ^1^H NMR spectra (Figures S1 and S4 and Figure 1), the target oligosiloxanes are the predominant products. The molecular weight of the byproduct siloxanes is well-defined, confirming that siloxane 1 and siloxane 2 are suitable for the intended application in this study.

### 2.2. Copolymerization of Oligosiloxanes and Polybutylene Terephthalate (**PBT**)

**Scheme 4:**
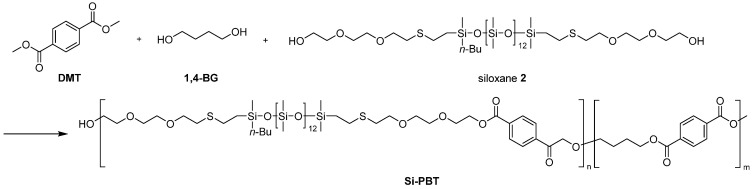
Synthesis of **Si-PBT**.

**Scheme 5:**
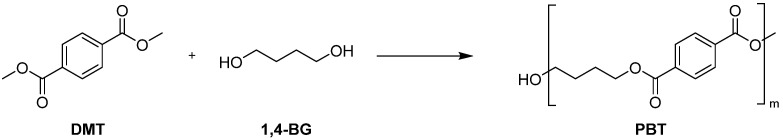
Synthesis of **PBT**.

The copolymerization of oligosiloxanes and polybutylene terephthalate (**PBT**) was performed in two steps. The first step involved a transesterification reaction, in which oligosiloxane, 1,4-butanediol (1,4-BG) and dimethyl terephthalate (DMT) reacted to form copolyester oligomer (Scheme 4 and Scheme 5). In the case of siloxane 1, phase separation occurred between 1 and 1,4-BG. The polarity of siloxane 1 was too low, causing the reaction melt to separate into two distinct phases. As a result, the subsequent polymerization step encountered an issue where the expansion torque did not increase sufficiently during the reaction. This is presumed to be due to the difficulty of siloxane’s reactive sites encountering those of DMT during transesterification, leading to a large amount of unreacted siloxane remaining in the siloxane phase prior to the second step. In contrast, siloxane 2 had sufficiently high polarity to achieve uniform transesterification reaction.

The second step was polymerization reaction, in which the oligomers extended their chain length to form polymers. As the degree of polymerization increased, the viscosity of the melt rose, leading to a higher expansion torque. When siloxane 1 was used, the stirring torque did not increase, and the copolyester resin synthesis failed. In contrast, with siloxane 2, the stirring torque increased to the expected value, and the copolyester resin **Si-PBT** was successfully obtained. This indicates that newly developed siloxane 2 has a higher affinity for polyester and is more effective in copolymerization compared to siloxane 1. For comparison, a **PBT** resin was also synthesized using the same method as for **Si-PBT**, but without using siloxane. The intrinsic viscosities and molecular weights of **Si-PBT** and **PBT** are shown in Table 1. Both materials were found to have a sufficient degree of polymerization for property evaluation.

**Figure 2:**
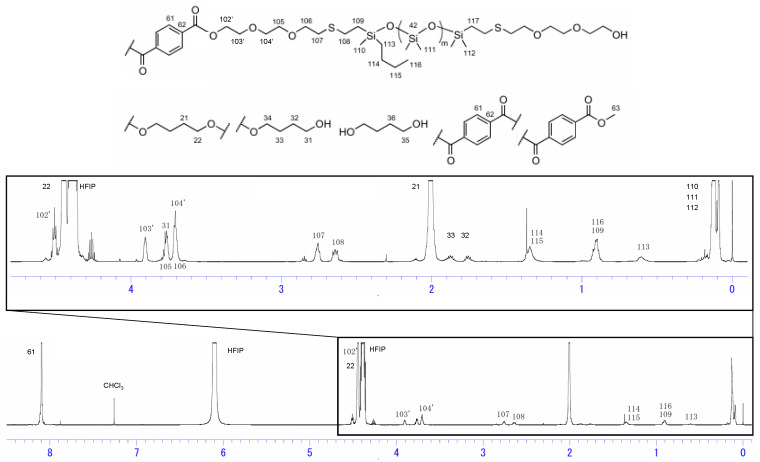
^1^H NMR spectra of **Si-PBT**.

The ^1^H NMR spectra of siloxane **2** and **Si-PBT** are shown in Figure 1 and Figure 2. In Figure 2, the disappearance of the 3.73 ppm peak assigned to H102, along with the appearance of the 4.50 ppm peak (H102′) and the 3.90 ppm peak (H103′), indicates that copolymerization between siloxane 2 and **PBT** was successfully achieved. The composition ratio of repeating units in **Si-PBT**, calculated from both the monomer feed ratio and the ratio determined by ^1^H NMR analysis, is shown in Table 2. These results confirm that no decomposition or volatilization of siloxane occurred, and that copolymerization proceeded successfully while maintaining the intended composition ratio.

The results of the polymerization experiment revealed that introducing highly polar oxyethylene groups at both termini of siloxane enhances its affinity for polyester, leading to the formation of a uniform copolymer.

### 2.3. Thermal Properties of Oligosiloxanes and Copolyesters

To evaluate the thermal stability of monomers and polymers, thermogravimetric analysis (TGA) measurements were conducted. From the TGA analysis of monomer 2, the temperature at which 3% weight loss (T_d3_) occurred was found to be 284.3 °C (Table 3), indicating that it can withstand the polymerization process temperature of 240 °C without decomposition.

**Figure 3:**
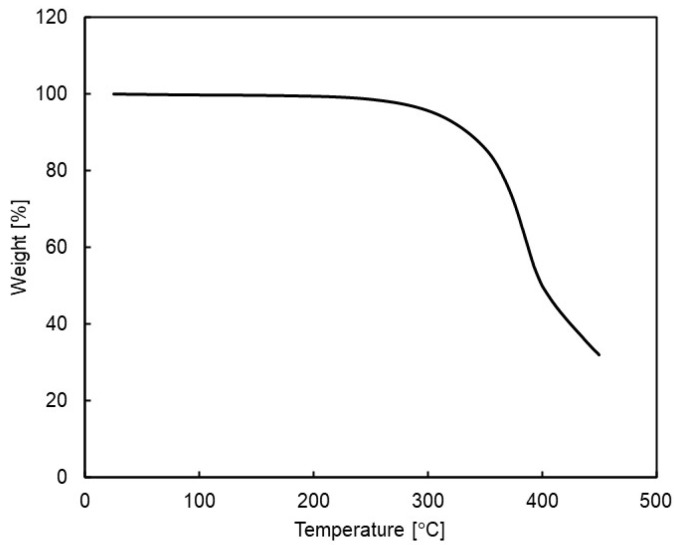
Thermogravimetric analysis (TGA) for siloxane **2** under nitrogen atmosphere.

The TGA analysis of the polymer showed no significant difference in weight loss behavior between **Si-PBT** and **PBT** under either nitrogen or air (Figure 3 and Table 4). This indicates that the introduction of a siloxane backbone into **PBT** does not compromise its inherent high thermal resistance. Consequently, **Si-PBT** can be considered a viable material for electrical components and molded parts, where polyester has traditionally been used for its heat resistance.

**Figure 4:**
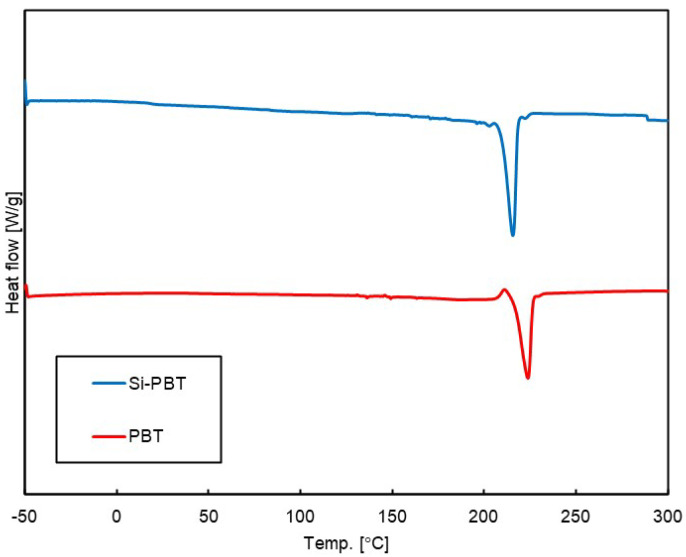
DSC curves of **Si-PBT** and **PBT** in secondary heating process.

Differential scanning calorimetry (DSC) measurements were conducted to investigate phase transition behavior under thermal conditions. The DSC curves of the polymers are shown in Figure 4, and the phase transition temperatures and enthalpies are listed in Table 5. Compared to **PBT**, the melting temperature of the crystalline domain in **Si-PBT** is shifted to a lower value, suggesting that the incorporation of the siloxane backbone into **PBT** disrupts its crystalline phase. In fact, the absolute crystallization enthalpy of **Si-PBT** is 34.7 J/g, which is lower than that of **PBT** (52.3 J/g). This further supports the hypothesis that the introduction of the siloxane backbone inhibits crystallization. This inhibition indicates that siloxane and polyester are miscible on the micrometer scale.

In this context, the term ‘crystalline phase’ refers to the macroscopic orientation of crystalline domains in polymeric materials. Unlike low-molecular-weight crystals, the degree of crystallinity is relatively low, estimated to be approximately 40% [21,22,23,24], making it challenging to obtain sufficient X-ray diffraction for structural observation.

### 2.4. Surface Property of Copolyester

To evaluate the impact of siloxane copolymerization on the surface properties of polyester, surface contact angle measurements were conducted on **Si-PBT** and **PBT**. The samples were prepared as films using heat press molding. The results are presented in Table 6. **Si-PBT** exhibited higher contact angles for all probe liquids compared to **PBT**, indicating improved water repellency and antifouling properties.

**Figure 5:**
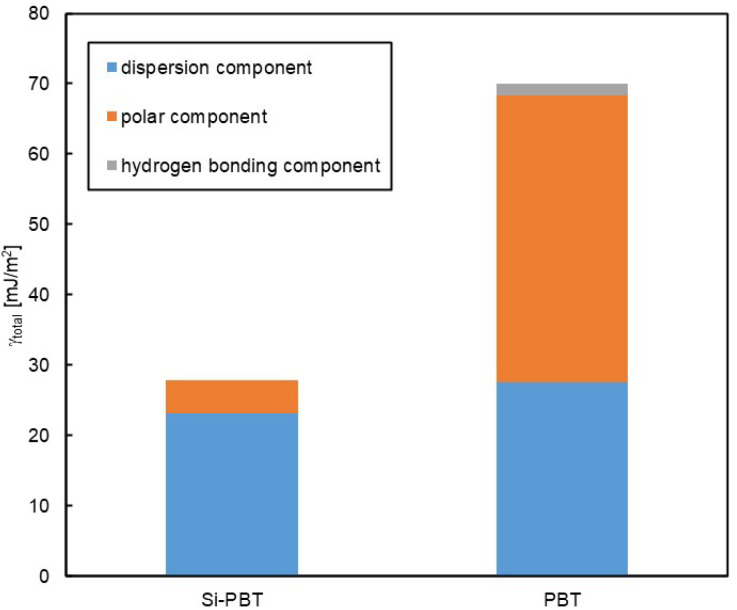
Surface free energy of **Si-PBT** and **PBT**.

To conduct a more detailed analysis, surface free energy component analysis was performed using the Kitazaki–Hata theory [25]. The results are presented in Table 7 and Figure 5. According to this theory, the surface free energy (*γ*) is divided into three components: the dispersion component (*γ*_d_), the polar component (*γ*_p_), and the hydrogen bonding component (*γ*_h_). In **Si-PBT**, a significant reduction in the polar component was observed. This suggests that the siloxane backbone, which has lower polarity than polyester, is preferentially distributed to the surface, thereby reducing surface polarity and lowering the total surface free energy. As intended, the incorporation of the siloxane backbone successfully reduced the surface energy of the polyester.

### 2.5. Impact Resistance of Copolyester

**Figure 6:**
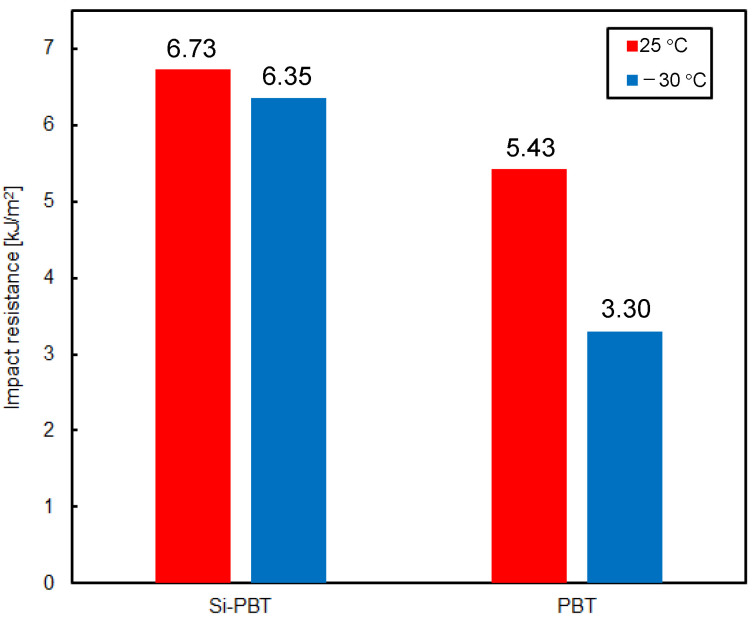
Impact resistance of **Si-PBT** and **PBT** at 25 °C or −30 °C.

Finally, the impact resistance of the polyester was evaluated to determine whether the introduction of the siloxane backbone leads to improvements. The results of the Charpy impact test [26,27], conducted at room temperature (25 °C) and low temperature (−30 °C), are shown in Figure 6. **Si-PBT** exhibited superior impact strength compared to **PBT**. Notably, at −30 °C, the impact strength of **Si-PBT** remained nearly unchanged from its room-temperature value. Such behavior is extremely rare and serves as a distinctive feature of **Si-PBT**.

Impact resistance generally arises from the conversion of kinetic energy into thermal energy at the molecular level within the polymer matrix. In this case, the flexible and spatially accommodating helical structure of siloxane is finely fragmented and uniformly dispersed throughout the polymer. This morphology prevents stress concentration at a single point and instead distributes the impact energy across the system, promoting efficient energy dissipation. The uniform dispersion and fine fragmentation of siloxane are further supported by the DSC results.

As shown in the DSC curve of **Si-PBT** in Figure 3, no thermally induced phase transition occurs between −30 °C and 25 °C. This thermal stability contributes to the excellent impact strength of **Si-PBT** at both room temperature and low temperatures.

## 3. Materials and Methods

### 3.1. Equipment

The Fourier transform nuclear magnetic resonance (NMR) spectra of oligosiloxanes were obtained using a JEOL JNM-ECA 400 (^1^H at 399.78 MHz) and a JEOL JNM-ECA 600 (^13^C at 150.91 MHz, ^29^Si at 119.24 MHz) NMR instrument. NMR spectra of **PBT**s were obtained using a Bruker AVANCE600 (^1^H at 600.1 MHz, ^13^C at 151 MHz, ^29^Si at 119.2 MHz) NMR instrument. For ^1^H NMR, chemical shifts were reported as δ units (ppm) relative to SiMe_4_ (TMS), and the residual solvent peaks were used as standards. For ^13^C NMR and ^29^Si NMR, chemical shifts were reported as δ units (ppm) relative to SiMe_4_ (TMS). The residual solvent peaks were used as standards, and the spectra were acquired with complete proton decoupling. Matrix-assisted laser desorption/ionization coupled time-of-flight (MALDI-TOF) mass analyses were performed by a Shimadzu (Kyoto, Japan) AXIMA Performance instrument, using 2,5-dihydroxybenzoic acid (2,5-DHBA) as the matrix and AgNO_3_ as the ion source. All used reagents were of analytical grade. Elemental analyses were performed at the Center for Material Research by Instrumental Analysis (CIA), Gunma University, Japan. TGA was performed using a Rigaku (Tokyo, Japan) thermogravimetric analyzer (Thermoplus TG-DTA8122). The investigations were carried out under nitrogen flow (50 mL min^−1^) or airflow (50 mL min^−1^) at a heating rate of 10 °C min^−1^. All samples were measured in a temperature range of 25 to 450 °C, with a 5 min hold at 450 °C. The weight loss and heating rate were continuously recorded during the experiment. Differential Scanning Calorimeter (DSC) was performed using a Mettler toledo (Columbus, OH, USA) DSC3. The investigations were carried out under nitrogen flow (40 mL min^−1^) at a heating rate of 10 °C min^−1^ and a cooling rate of 10 °C min^−1^. All samples were measured in a temperature range of −50 to 300 °C, with a 5 min hold at the end of each process; −50 °C and 300 °C. Contact angle measurements were performed using a Kyowa Interface Science Co. LTD. (Tokyo, Japan) CA-X150. The contact angles were observed 0.1 s after dropping at 25 °C and 50%RH. Impact tests were performed using a TOYOSEIKI (Tokyo, Japan) impact tester (D-CB). Impact resistance was evaluated using the notched Charpy test [26]. Gel permeation chromatography (GPC) was performed using a Tosoh Corporation (Tokyo, Japan) HLC-8220 instrument. Chloroform was used as a mobile phase at 40 °C. PLgel 10μ Mixed-B (7.5 mm I.D. × 30 cmL × 2) was chosen as the column. RI detector was used and the calibration was performed using monodisperse polystyrene. The viscosity measurements were performed using a Sentec Co., Ltd. (Osaka, Japan) fully automated viscometer DT553 (capillary type). A mixed solvent composed of phenol and 1,1,2,2-tetrachloroethane in a mass ratio of 1:1 was used. At 30 °C, the falling time of the test solution—either siloxane copolymerized **PBT** or **PBT** with a concentration of 1.0 g/dL—and the solvent alone was measured. The intrinsic viscosity was then calculated using the following equation:

**Intrinsic viscosity (dL/g) = ((1 + 4KH*η*_sp_)^0.5 − 1)/(2KH C)** (1)

where ***η*_sp_ = *η*/*η*_0_ − 1**, ***η*** represents the falling time of the sample solution, ***η*_0_** is the falling time of the solvent, **C** is the concentration of the siloxane copolymerized **PBT** or **PBT** in the sample solution (g/dL), and **KH** is Huggins’ constant, which was set to **0.33**.

### 3.2. General Considerations of Synthesis

The synthesis of oligosiloxanes was performed under an argon atmosphere using standard Schlenk techniques, unless otherwise noted. Polymerization reactions were performed under a nitrogen atmosphere. Tetrahydrofuran (THF) and toluene were dried using an mBRAUN solvent purification system. Trimethyltrivinylcyclotrisiloxane, hexamethylcyclotrisiloxane, chlorodimethylvinylsilane, 2,2′-azobis(isobutyronitrile)) (AIBN), tetrabutyl orthotitanate, and magnesium acetate were purchased from Tokyo Chemical Industry Co., Ltd. (Tokyo, Japan). Toluene, 2-mercaptoethanol, NaHCO_3_, CH_2_Cl_2_, Na_2_SO_4_, and magnesium acetate were purchased from FUJIFILM Wako Pure Chemical Corporation (Tokyo, Japan). *n*-Butyllithium (1.6 M in hexane) was purchased from KANTO CHEMICAL CO., INC. (Tokyo, Japan). 1,4-Butanediol was purchased from Mitsubishi Chemical Corporation (Tokyo, Japan). Dimethyl terephthalate was purchased from SK Chemicals Corporation (Gyeonggi, Korea). All reagents were used as received without further purification. 2-[2-(2-Mercapto Ethoxy)ethoxy]ethanol was synthesized using a modification of a known procedure [28].

### 3.3. Synthetic Procedures of Compounds

#### 3.3.1. Synthesis of PDMS-Vi

The ring-opening reaction conditions using *n*-butyllithium (*n*BuLi) were optimized based on those reported in the literature [29].

An argon-purged, three-necked, 100 mL round-bottom flask equipped with a stir bar was charged with trimethyltrivinylcyclotrisiloxane (Vi, Me-D3) (1.35 mL, 5.0 mmol) and dry toluene (1.50 mL). The mixture was cooled to 0 °C and *n*BuLi (1.6 M in hexane, 9.40 mL, 15.0 mmol) was added dropwise into the mixture under argon at 0 °C. After the addition, the reaction mixture was stirred for 30 min at 0 °C. Hexamethylcyclotrisiloxane (D3) (13.4 g, 60.0 mmol) was then added to the reaction mixture, followed by the addition of dry THF (2.50 mL, 30.8 mmol) as a polymerization promoter at room temperature. The reaction mixture was stirred for 5 h at room temperature, and then a slight excess of chlorodimethylvinylsilane (2.14 mL, 15.5 mmol) was added to terminate the polymerization. The solution was stirred overnight at room temperature and then washed three times with brine. The organic layer was evaporated by a rotary evaporator for 1.5 h at 35 °C, and then dried under vacuum at 60 °C for >3 h to afford PDMS-Vi (9.60 g, 8.58 mmol, 57% yield) as a transparent colorless liquid. The characterization data are listed in the Supporting Information.

#### 3.3.2. Synthesis of Siloxane **1**

The thiol-ene reaction conditions using 2,2′-azobis(isobutyronitrile) (AIBN) were optimized based on those reported in the literature [19,20,30].

An argon-purged Schlenk flask equipped with a stir bar was charged with AIBN (19.7 mg, 0.12 mmol) and anhydrous THF (10 mL). The solution was stirred for 5 min at room temperature and PDMS-Vi (6.21 mL, 5 mmol) was added. The mixture was heated to 40 °C and after stirring for 10 min, 2-mercaptoethanol (1.06 mL, 15 mmol) was added dropwise at 40 °C. The mixture was then heated to 60 °C and stirred at 60 °C for 24 h. After the reaction, the mixture was cooled to room temperature and quenched with saturated NaHCO_3_ aqueous solution (20 mL). The resulting mixture was extracted with CH_2_Cl_2_ three times, and the gathered organic layer was washed with water three times and brine two times, and dried over anhydrous Na_2_SO_4_. After filtration, the solvent was removed using a rotary evaporator to afford siloxane 1 (5.23 g, 4.10 mmol, 82% yield) as a slightly yellow, viscous oil. The characterization data are listed in the Supporting Information.

#### 3.3.3. Synthesis of Siloxane **2**

An argon-purged Schlenk flask equipped with a stir bar was charged with AIBN (19.7 mg, 0.12 mmol) and anhydrous THF (10 mL). The solution was stirred for 5 min at room temperature and PDMS-Vi (6.21 mL, 5 mmol) was added. The mixture was heated to 40 °C and after stirring for 10 min, 2-[2-(2-Mercapto Ethoxy)ethoxy]ethanol (2.15 mL, 15 mmol) was added dropwise at 40 °C. The mixture was then heated to 60 °C and stirred at 60 °C for 24 h. After the reaction, the mixture was cooled to room temperature and quenched with saturated NaHCO_3_ aqueous solution (20 mL). The resulting mixture was extracted with CH_2_Cl_2_ three times, and the gathered organic layer was washed with water three times and brine two times, and dried over anhydrous Na_2_SO_4_. After filtration, the solvent was removed using a rotary evaporator to afford siloxane 2 (5.90 g, 4.01 mmol, 81% yield) as a slightly yellow, viscous oil. The characterization data are listed in the Supporting Information.

#### 3.3.4. Synthesis of **Si-PBT**

**Figure 7:**
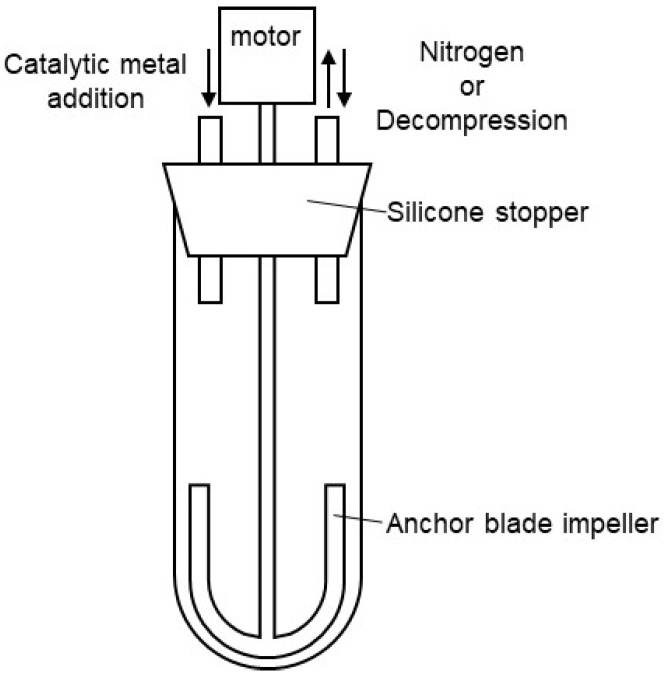
Grass reactor for polyester polymerization.

A nitrogen-purged Schlenk tube, as shown in Figure 7, was charged with dimethyl terephthalate (DMT) (15.9 g, 0.08 mol), 1,4-butanediol (1,4-BG) (8.12 g, 0.09 mol), and siloxane 2 (7.71 g, 8.2 mmol), and then heated to 150 °C. Tetrabutyl orthotitanate (6.0 wt% in 1,4-BG solution, 0.10 g, 234 ppm) was added to the melted mixture under a nitrogen atmosphere at 150 °C. After the addition, the mixture was heated to 210 °C over 105 min, and stirred at 210 °C for 60 min. The transesterification reaction was completed and 5.2 mL of methanol was distilled off. Then, magnesium acetate (10.0 wt% in 1,4-BG solution, 0.11 g, 423 ppm) and tetrabutyl orthotitanate (6.0 wt% in 1,4-BG solution, 0.18 g, 433 ppm) were added to the mixture under a nitrogen atmosphere at 210 °C. After the addition, the reaction system was gradually decompressed to 1 torr over 90 min. A total of 5 min after the start of decompression, the temperature was increased to 240 °C over the course of 90 min. Continued stirring at 240 °C for an additional 90 min afforded silicone-copolymerized polybutylene terephthalate (**Si-PBT**) as a transparent viscous liquid. Although an increase in torque was observed during the polymerization reaction, the Weissenberg effect [31] caused polymer entanglement around the stirring blades, preventing the acquisition of an accurate time–torque profile. Upon removal from the Schlenk tube, the product solidified into a crystalline white solid (19.8 g).

#### 3.3.5. Synthesis of **PBT**

A nitrogen-purged Schlenk tube, as shown in Figure 7, was charged with dimethyl terephthalate (DMT) (132.27 g, 0.68 mol) and 1,4-butanediol (1,4-BG) (73.66 g, 0.82 mol) was heated to 150 °C. Tetrabutyl orthotitanate (6.0 wt% in 1,4-BG solution, 0.59 g, 234 ppm) was added to the melted mixture under a nitrogen atmosphere at 150 °C. After the addition, the mixture was heated to 210 °C over 105 min and stirred at 210 °C for 60 min. The transesterification reaction was completed and 50 mL of methanol was distilled off. Then, magnesium acetate (10.0 wt% in 1,4-BG solution, 0.64 g, 423 ppm) and tetrabutyl orthotitanate (6.0 wt% in 1,4-BG solution, 1.08 g, 433 ppm) were added to the mixture under a nitrogen atmosphere at 210 °C. After the addition, the reaction system was gradually decompressed to 1 torr over 90 min. A total of 5 min after the start of decompression, the temperature was increased to 240 °C over the course of 90 min. Continued stirring at 240 °C for an additional 90 min afforded polybutylene terephthalate (**PBT**) as a transparent viscous liquid. Upon removal from the Schlenk tube, the product solidified into a crystalline white solid (121.2 g).

## 4. Conclusions

In conclusion, a novel siloxane-based comonomer for polyester was successfully designed and synthesized to improve the surface hydrophobicity and low-temperature impact resistance. By introducing siloxane into the polyester structure, we aimed to enhance water resistance while improving flexibility and mechanical durability in cold environments. The findings confirmed that a properly engineered siloxane molecular structure promotes uniform copolymerization with polyester, effectively overcoming previous challenges related to phase separation and compatibility issues.

Analysis of the synthesized copolymers demonstrated that siloxane incorporation significantly increased surface hydrophobicity, as evidenced by higher contact angles. Additionally, low-temperature impact tests indicated that the presence of siloxane contributed to enhanced flexibility, reducing brittleness and improving performance under sub-zero conditions.

The results of this study highlight the potential of molecular-level polymer design in achieving high-performance materials without the need for additional coatings. This research provides valuable insights into the development of durable, hydrophobic polyester materials suitable for various applications. Future work will focus on further optimizing siloxane composition and distribution within the polymer to achieve even greater functionality and performance.

## Figures and Tables

**Table 1:** The intrinsic viscosities and molecular weights of **Si-PBT** and **PBT**.

Polymer	*η*_red_ [dL/g] ^1^	Mn [g/mol] ^2^	Mw [g/mol] ^2^	PDI ^2^
**Si-PBT**	0.89	1.5 × 10^4^	6.0 × 10^4^	3.9
**PBT**	1.08	1.7 × 10^4^	4.4 × 10^4^	3.5

^1^ Calculated from viscosity measurements. ^2^ Calculated from GPC measurements.

**Table 2:** The composition ratio of repeating units in **Si-PBT**.

	DMT [mol%]	1,4-BG [mol%]	Siloxane 2 [mol%]
Feed	100	95	5
^1^H NMR ^1^	100	93	4 ^1^

^1^ Assuming that the degree of polymerization of the siloxane portion in the raw material siloxane **2** and the siloxane copolymerized polyester is the same, quantification was performed using the H113 signals.

**Table 3:** The decomposition temperatures [°C] of siloxane **2** under nitrogen atmosphere.

	*T*_d3_	*T*_d5_	*T*_d10_
Siloxane **2**	284.3	306.2	355.2

**Table 4:** The decomposition temperatures [°C] of **Si-PBT** and **PBT** under nitrogen and air atmosphere.

	N_2_ Atmosphere			Air Atmosphere		
	*T*_d3_	*T*_d5_	*T*_d10_	*T*_d3_	*T*_d5_	*T*_d10_
**Si-PBT**	330.6	344.6	359.0	321.9	335.1	350.7
**PBT**	359.9	368.3	376.3	328.7	354.0	367.4

**Table 5:** Phase transition temperature and enthalpy of **Si-PBT** and **PBT** ^1^.

	*T*_m_ [°C]	*Δ*H(*T*_m_) [J/g]
**Si-PBT**	216.4	−34.7
**PBT**	223.8	−52.3

^1^ The data for the secondary heating process are illustrated.

**Table 6:** Contact angles [°] of **Si-PBT** and **PBT** against each proves ^1^.

	Water	*n*-Hexadecane	CH_2_I_2_
**Si-PBT**	113.3	15	52.4
**PBT**	77.8	5.5	23.8

^1^ The shape was measured 0.1 s after the application of the liquid.

**Table 7:** Surface free energy *γ* [mJ/m^2^] of **Si-PBT** and **PBT**.

	*γ*_d_	*γ*_p_	*γ*_h_	*γ*_total_
**Si-PBT**	23.2	4.6	0.0	27.8
**PBT**	27.5	40.8	1.7	70.0

## Data Availability

All data and materials described in this work are available in this article or in the Supplementary Materials.

## Supplementary Materials

The following supporting information can be downloaded at: https://www.mdpi.com/article/10.3390/molecules30132775/s1, characterization data for PDMS-Vi, siloxane 1 and 2, **Si-PBT**, ^1^H, ^13^C, ^29^Si NMR spectra of PDMS-Vi, siloxane **1** and **2** (Figures S1–S10); MALDI-TOF MS spectra of PDMS-Vi, siloxane **1** and **2** (Figures S11–S13); thermogravimetric analysis (TGA) spectra for **Si-PBT** and **PBT** (Figure S14).
