# Supplementary material for: Functionalization of Oligosiloxane for Polyester Comonomer
Source: Molecules. 2025 Jun 27;30(13):2775. doi: 10.3390/molecules30132775 (PMC12250609; doi:10.3390/molecules30132775)
Supplement: Supplementary file 1 [file molecules-30-02775-s001.zip › molecules-3683767-supplementary.pdf]

Supplementary Materials

Functionalization of oligosiloxane for polyester comonomer

Satoru Saotome [1,2], Jiaorong Kuang [1], Reina Akashi [1], Momoko Takahashi [1], Yujia Liu [1,*], Takayuki Iijima [2] and Masafumi Unno [1,*]

[1] Department of Chemistry and Chemical Biology, Gunma University, 1-5-1 Tenjin-cho, Kiryu 376-8515, Japan
[2] Polyester Laboratory, Polymers Research & Development Center, Mitsubishi Chemical Corporation, 1, Toho-cho, Yokkaichi-shi, Mie, 510-8530, Japan
* Correspondence: yliu@gunma-u.ac.jp (Y.L.); unno@gunma-u.ac.jp (M.U.); Tel.: +81-27-730-1230 (M.U.)

1. Characterization date for compounds

PDMS-Vi

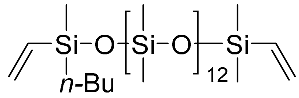

PDMS-Vi

^{1}H NMR (399.78 MHz, CDCl$_3$): δ = 0.02-0.14 (m, 81H), 0.57-0.61 (m, 2H), 0.87 (t, J = 6.8 Hz, 3H), 1.29-1.32 (m, 4H), 5.67 (dd, J = 4.0, 1.2, 1H), 5.75 (dd, J = 4.0, 1.6, 1H), 5.94 (dd, J = 6.4, 4.4 Hz, 1H), 5.95 (dd, J = 6.0, 4.0, 1H), 6.05-6.16 (m, 2H) ppm

^{29}Si NMR (119.24 MHz, CDCl$_3$): δ = –21.53, –21.34, –20.67, –20.35, –3.57, –3.47 ppm

^{13}C NMR (150.91 MHz, CDCl$_3$): δ = –1.47, 0.33, 1.12, 1.24, 13.86, 16.74, 25.34, 26.42, 131.70, 132.03, 138.62, 139.42 ppm

MALDI-TOF MS (m/z): 1138.98 ([M+Na]$^+$, calcd 1139.35)

Elemental analysis: Calcd for $C_{35}H_{96}O_{13}Si_{14}$: C, 37.59; H, 8.65. Found: C, 37.49; H, 8.61.

Siloxane **1**

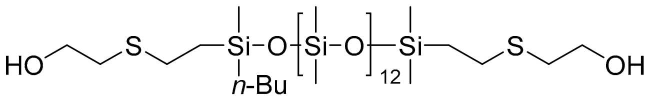

siloxane **1**

^{1}H NMR (399.78 MHz, CDCl$_3$): δ = 0.04-0.10 (m, 81H), 0.53-0.57 (m, 2H), 0.85-0.91 (m, 7H), 1.24-1.34 (m, 3H), 2.16-2.25 (m, 2H), 2.57 (td, J = 8.8, 1.6, 4H), 2.73 (t, J = 6.0 Hz, 4H), 3.72 (q, J = 6.0, 4H) ppm

^{29}Si NMR (119.24 MHz, CDCl$_3$): δ = –21.45, –21.37, –20.74, –20.37, 6.30, 6.38 ppm

^{13}C NMR (150.91 MHz, CDCl$_3$): δ = –1.45, 0.27, 1.12, 1.23, 13.85, 16.56, 17.90, 19.17, 25.33, 26.45, 26.50, 35.17, 60.10 ppm

MALDI-TOF MS (m/z): 1295.09 ([M+Na]$^+$, calcd 1295.38)

Elemental analysis: Calcd for $C_{39}H_{108}O_{15}S_2Si_{14}$: C, 36.75; H, 8.45; S, 5.03. Found: C, 36.95; H, 8.72; S, 5.46.

Siloxane **2**

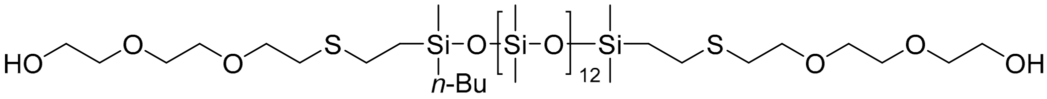

siloxane **2**

^{1}H NMR (399.78 MHz, CDCl$_3$): δ = 0.00-0.20 (m, 81H), 0.54 (t, J = 5.9, 2H), 0.85-0.91 (m, 7H), 1.24-1.31 (m, 4H), 2.41-2.48 (m, 2H), 2.59 (td, J = 8.8, 2.0, 4H), 2.72 (t, J = 7.2 Hz, 4H), 3.59-3.67 (m, 16H), 3.71-3.74 (m, 4H) ppm

^{29}Si NMR (119.24 MHz, $CDCl_3$): δ = –21.39, –20.83, –20.45, 6.40 ppm

^{13}C NMR (150.91 MHz, $CDCl_3$): δ = 0.27, 1.10, 1.25, 13.87, 16.57, 17.89, 19.16, 25.34, 26.46, 27.50, 31.27, 61.88, 70.40, 70.49, 70.94, 72.58 ppm

MALDI-TOF MS (m/z): 1472.17 ($[M+Na]^+$, calcd 1471.48)

Elemental analysis: Calcd for $C_{47}H_{124}O_{19}S_2Si_{14}$: C, 38.91; H, 8.62, S; 4.42. Found: C, 38.66; H, 8.73; S, 2.57.

2.1. H, ^{13}C, ^{29}Si NMR Spectra for compounds

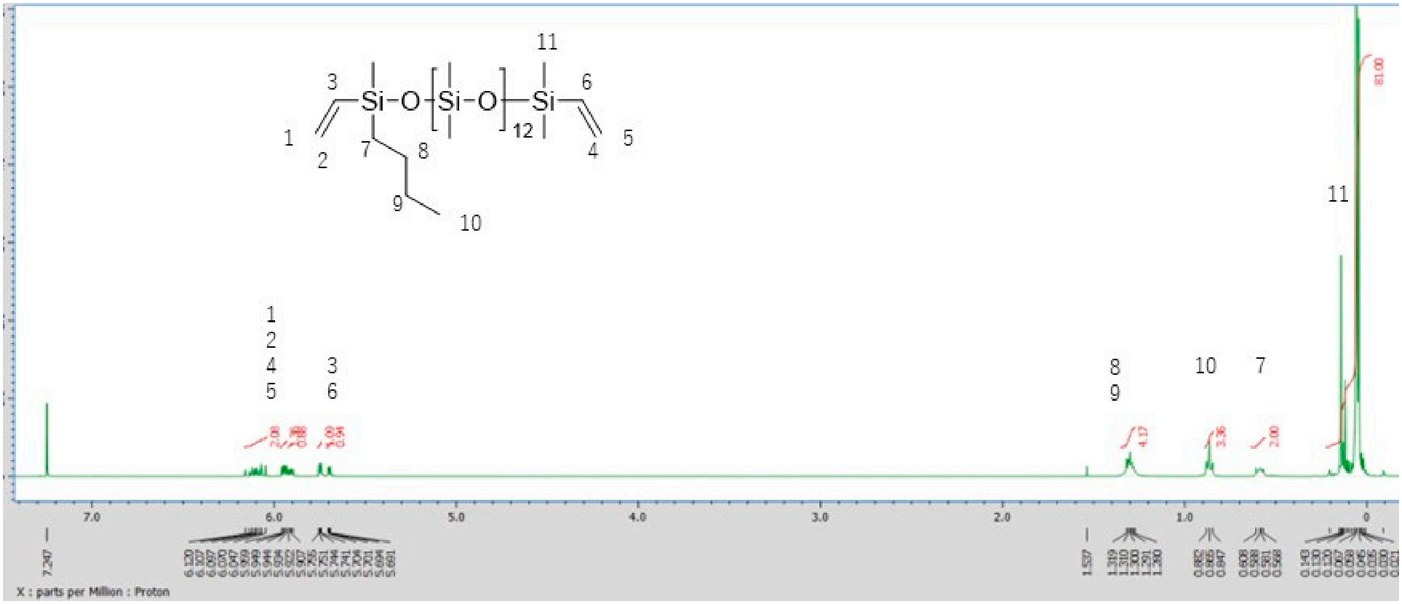

Figure S1. ^1H NMR spectrum for PDMS-Vi.

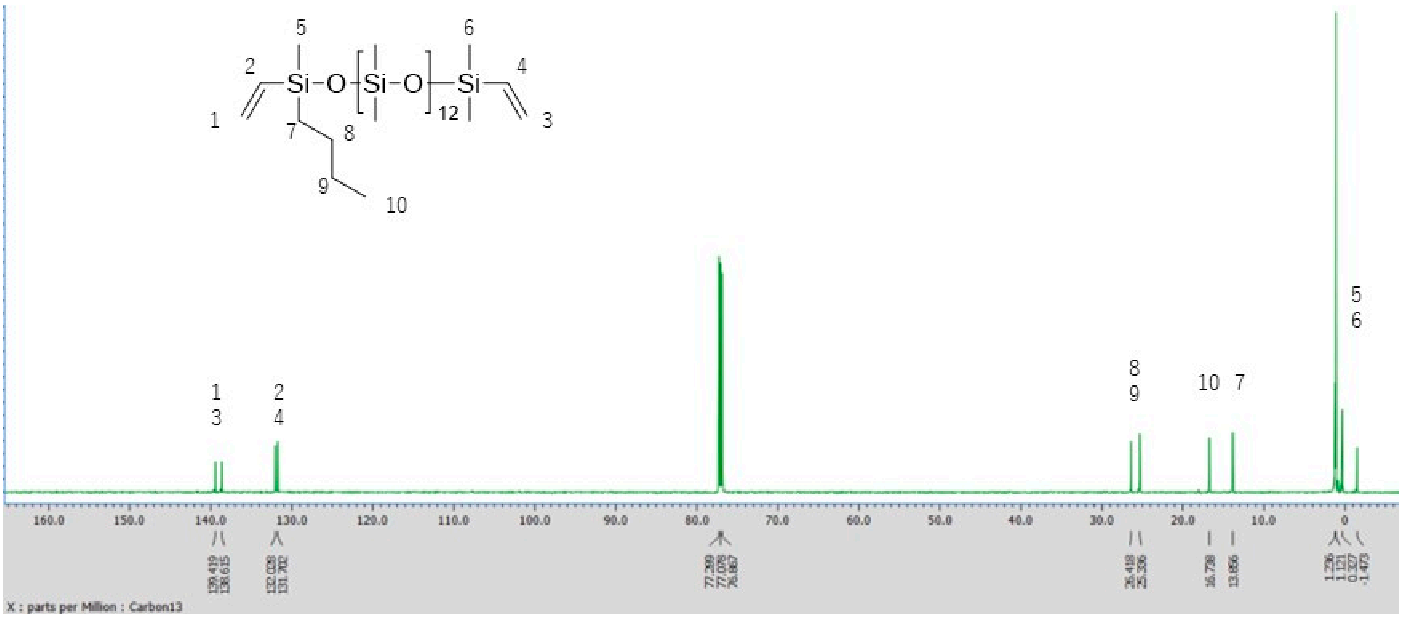

Figure S2. ^{13}C NMR spectrum for PDMS-Vi.

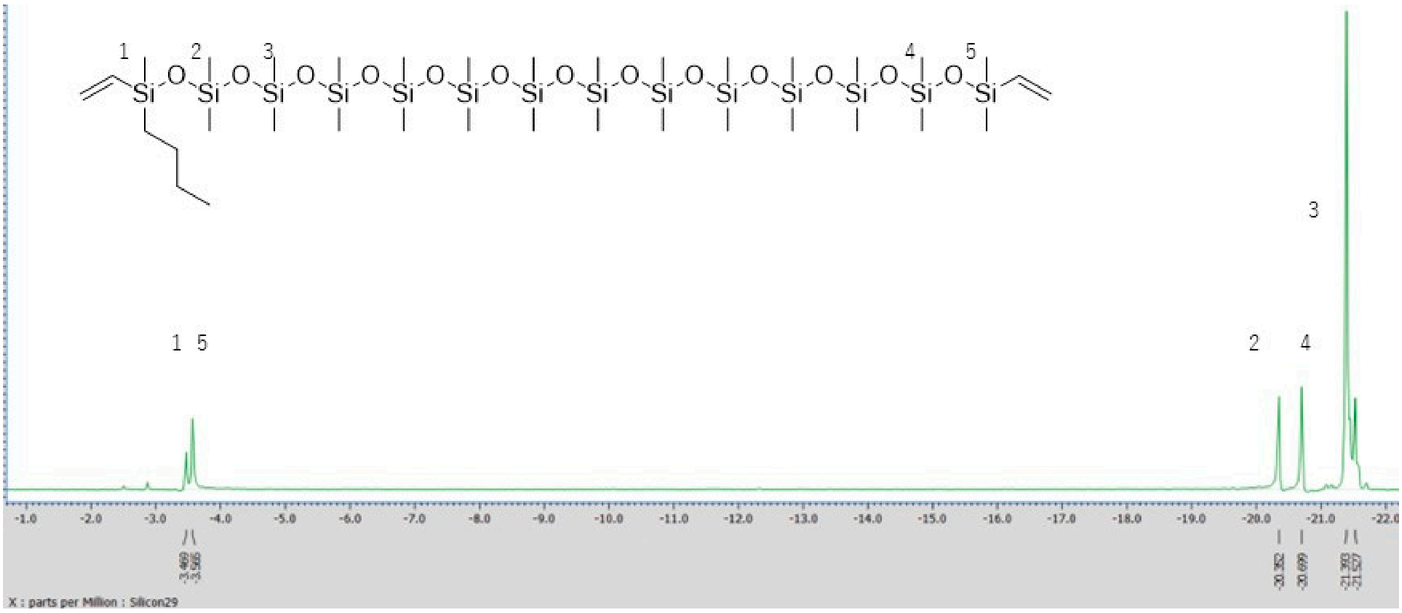

Figure S3. ^{29}Si NMR spectrum for PDMS-Vi.

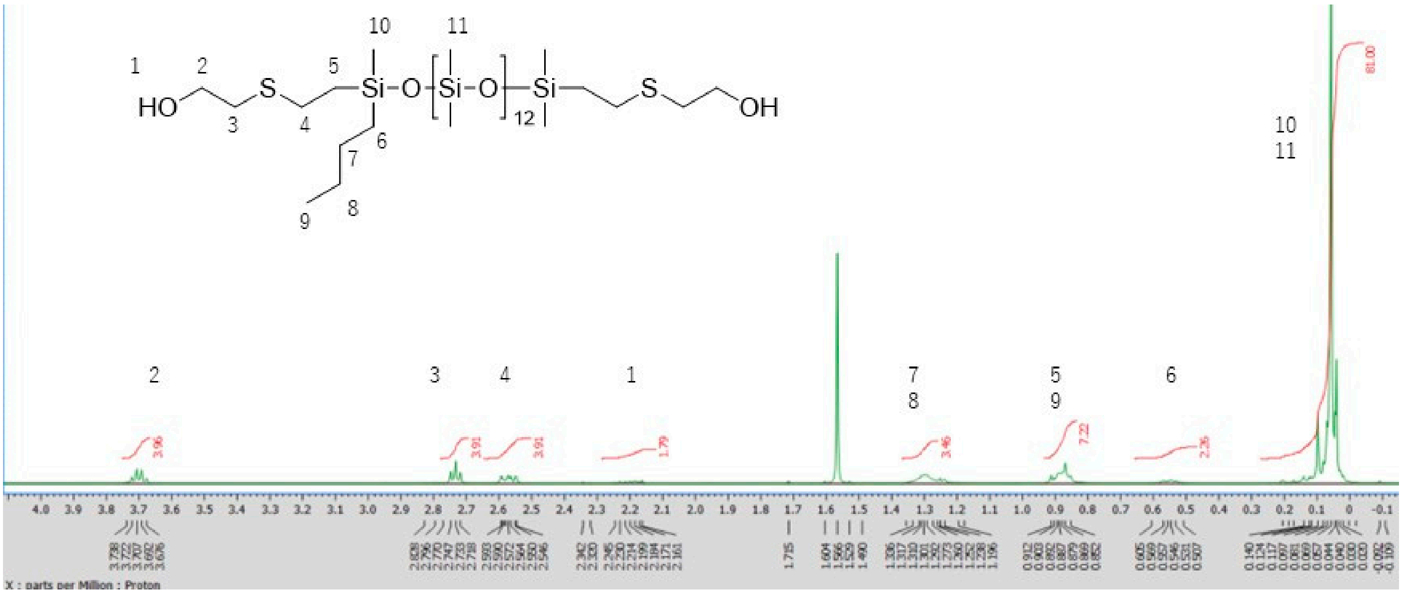

Figure S4. ^{1}H NMR spectrum for siloxane 1.

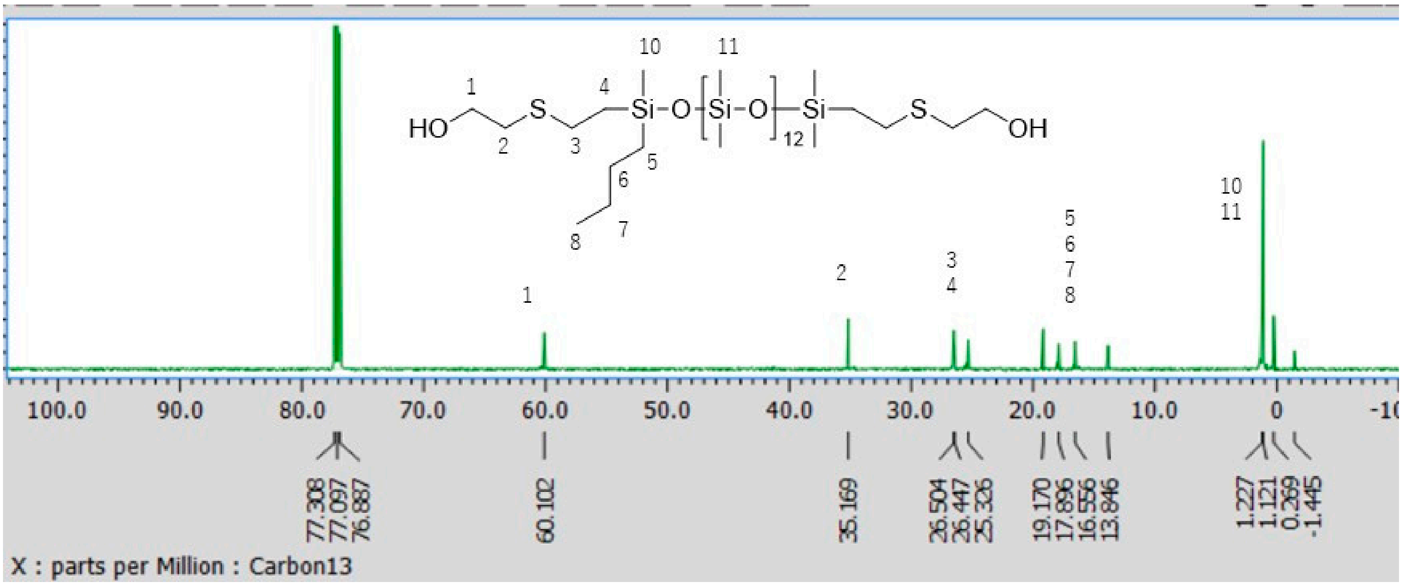

Figure S5. ^{13}C NMR spectrum for siloxane 1.

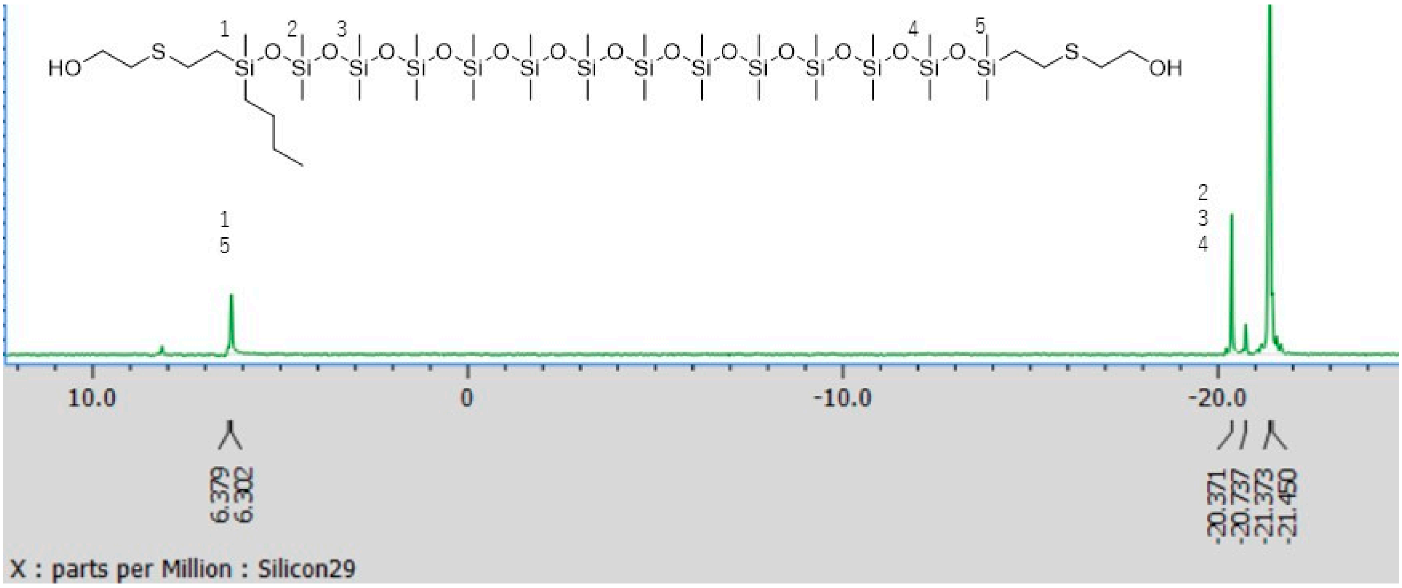

Figure S6. ^{29}Si NMR spectrum for siloxane 1.

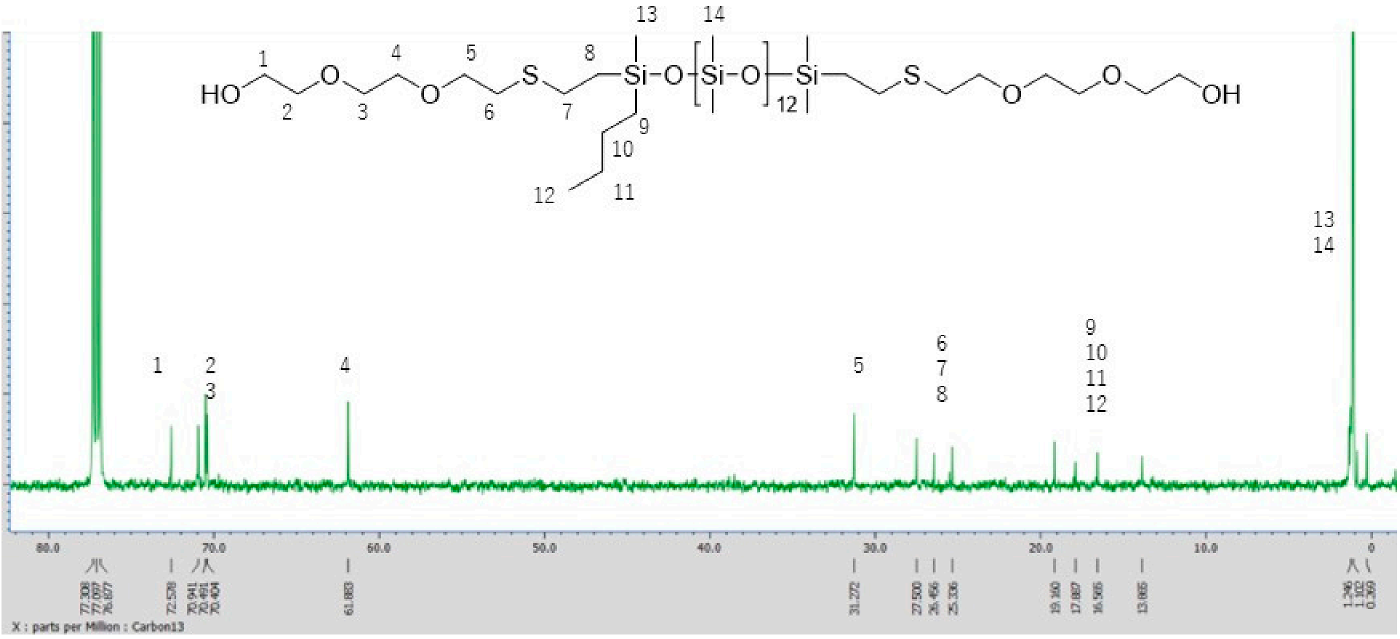

Figure S7. ^{13}C NMR spectrum for siloxane 2.

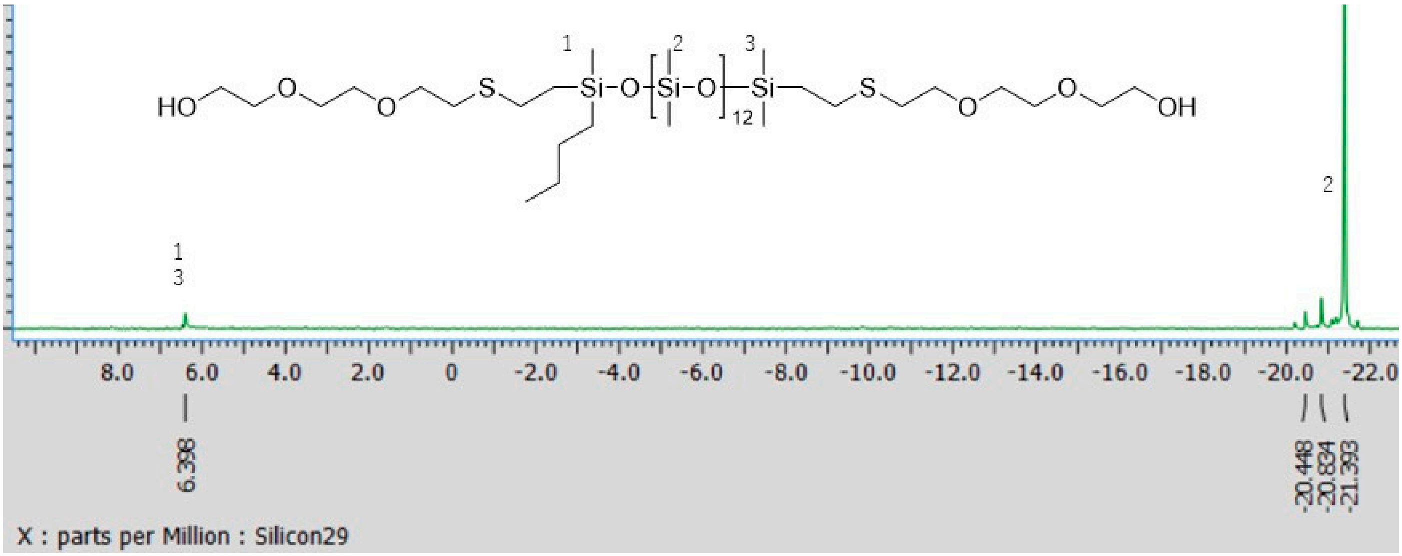

Figure S8. ^{29}Si NMR spectrum for siloxane 2.

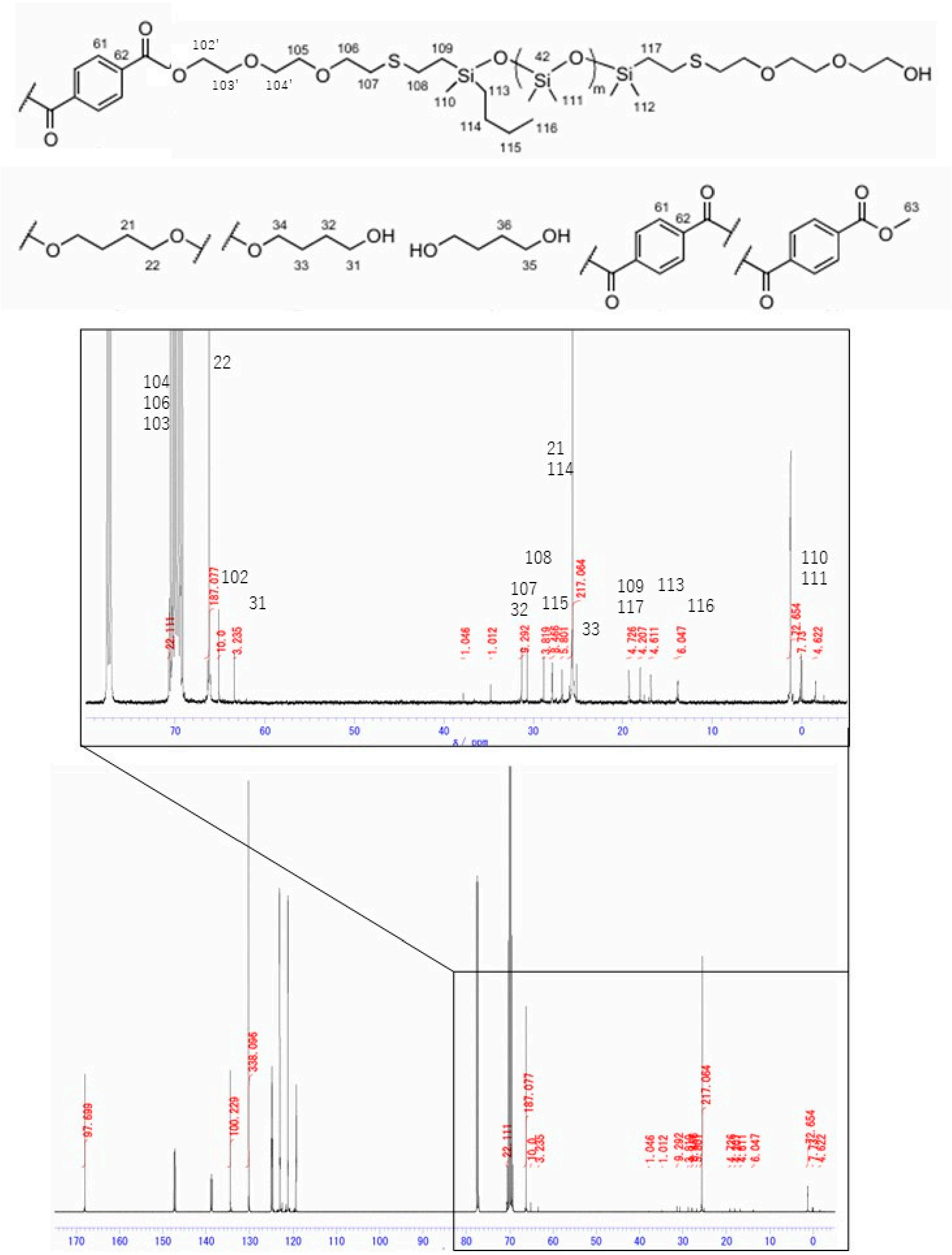

Figure S9. 13C NMR spectrum for Si-PBT.

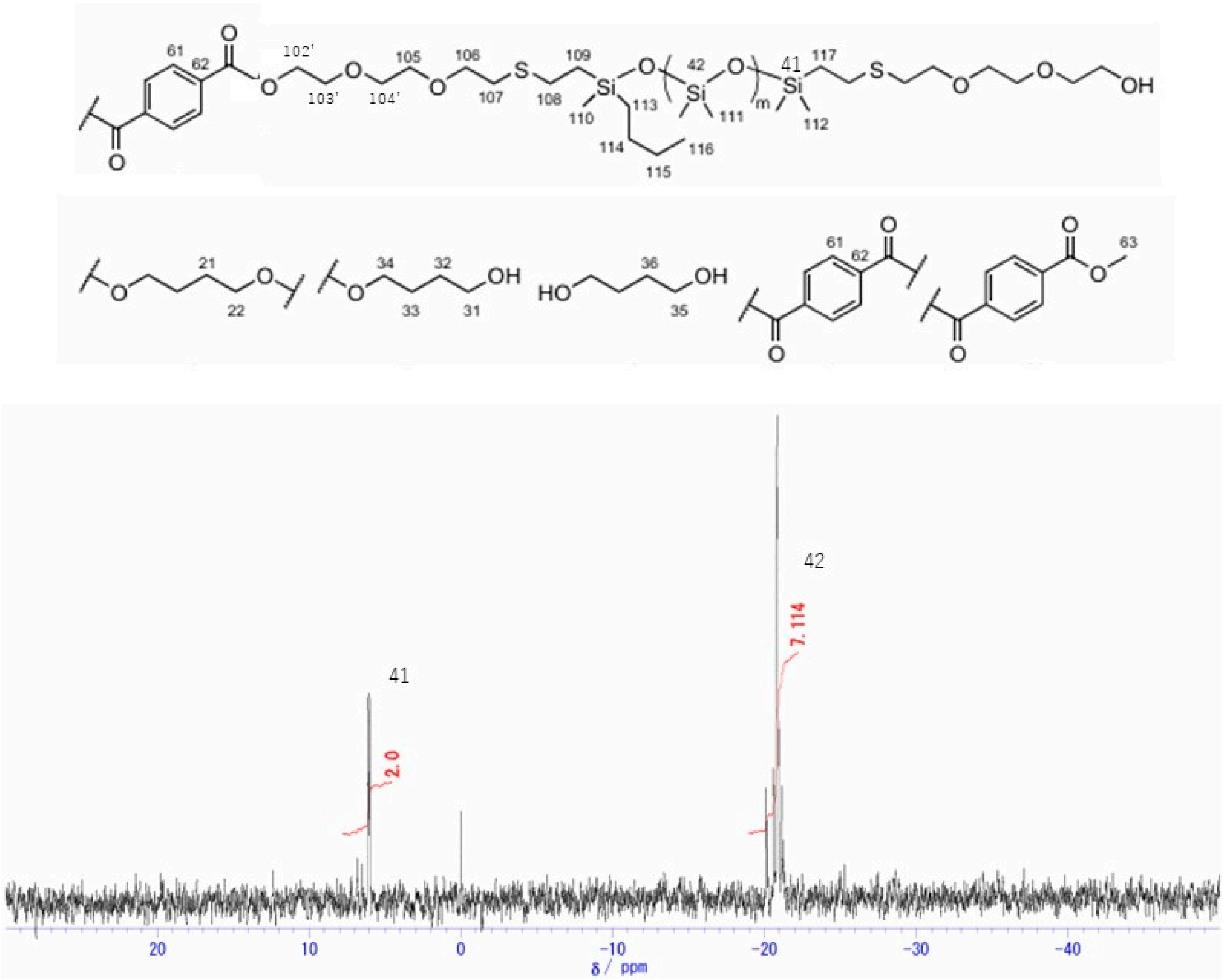

Figure S10. ^{29}Si NMR spectrum for Si-PBT.

3. MALDI-TOF MS Spectra

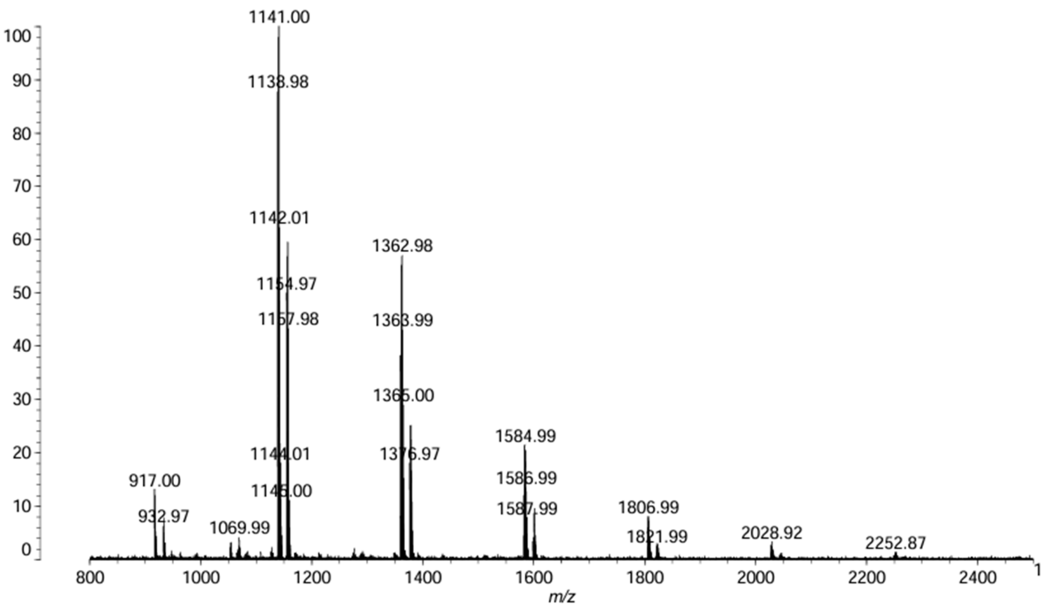

Figure S11. MALDI-TOF MS spectrum for **PDMS-Vi**.

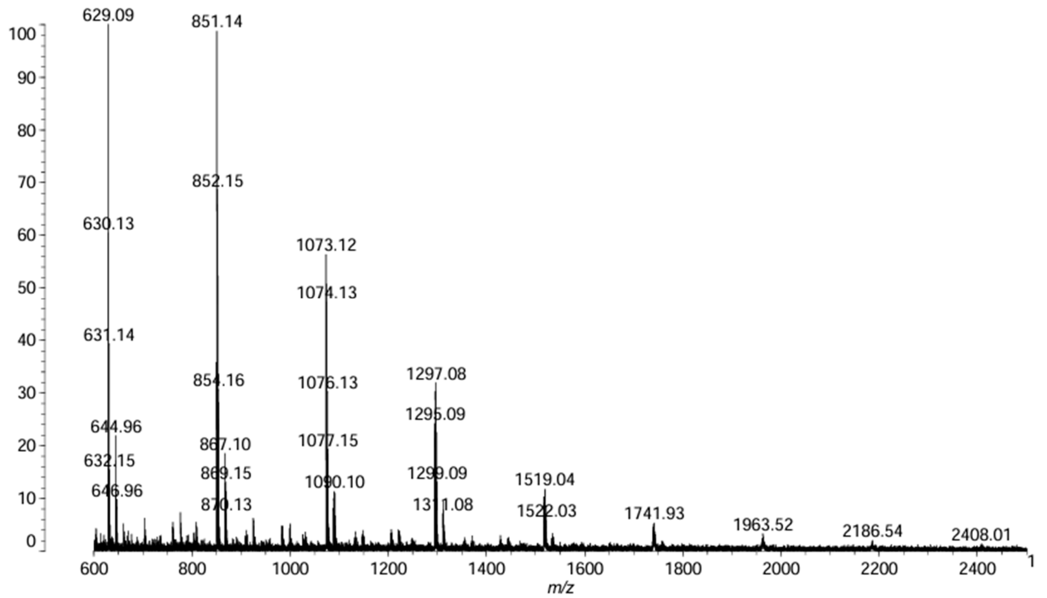

Figure S12. MALDI-TOF MS spectrum for **siloxane 1.**

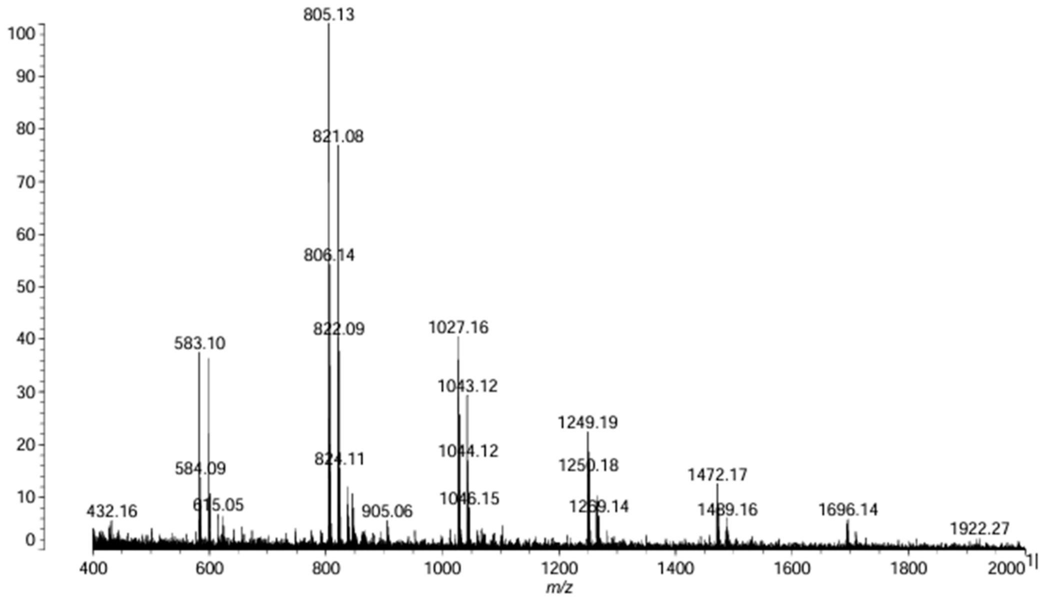

Figure S13. MALDI-TOF MS spectrum for **siloxane 2.**

4. Thermogravimetric analysis (TGA) for Si-PBT and PBT

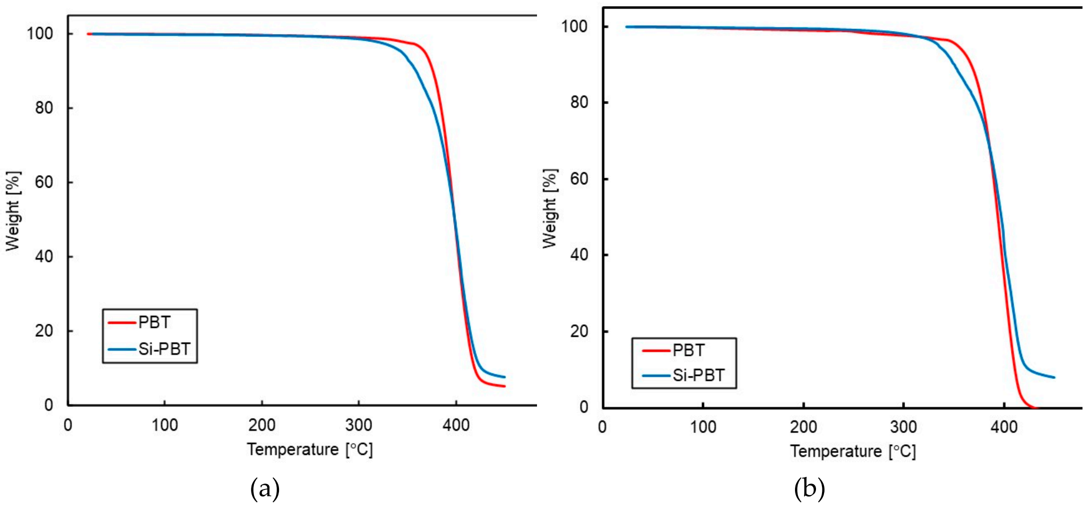

Figure S14. Thermogravimetric analysis (TGA) for **Si-PBT** and **PBT** under (a) nitrogen or (b) air atmosphere.
